# Increase in malaria prevalence and age of at risk population in different areas of Gabon

**DOI:** 10.1186/1475-2875-12-3

**Published:** 2013-01-02

**Authors:** Denise P Mawili-Mboumba, Marielle K Bouyou Akotet, Eric Kendjo, Joseph Nzamba, Mathieu Owono Medang, Jean-Romain Mourou Mbina, Maryvonne Kombila

**Affiliations:** 1 Department of Parasitology-Mycology, Faculty of Medicine, Université des Sciences de la Santé, Libreville BP 4009, Gabon

**Keywords:** *Plasmodium falciparum*, Children, Fever, Age, Gabon

## Abstract

**Background:**

Following the deployment of new recommendations for malaria control according to the World Health Organization, an estimation of the real burden of the disease is needed to better identify populations at risk and to adapt control strategies. The aim of the present study was to estimate the clinical burden of malaria among febrile children aged less than 11 years, before and after six-year of deployment of malaria control strategies in different areas of Gabon.

**Methods:**

Cross-sectional surveys were carried out in health care facilities at four locations: two urban areas (Libreville and Port-Gentil), one semi-urban area (Melen) and one rural area (Oyem), between 2005 and 2011. Febrile paediatric patients, aged less than 11 years old were screened for malaria using microscopy. Body temperature, history of fever, age, sex, and location were collected.

**Results:**

A total of 16,831 febrile children were enrolled; 78.5% (n=13,212) were less than five years old. The rate of *Plasmodium falciparum*-infection was the lowest in Port-gentil (below 10%) and the highest at Oyem (above 35%). Between 2005 and 2008, malaria prevalence dropped significantly from 31.2% to 18.3%, followed by an increase in 2011 in Libreville (24.1%), Port-Gentil (6.5%) and Oyem (44.2%) (*p*<0.01). Median age among the infected patients increased throughout the study period reaching 84 (60–108) months in Libreville in 2011 (*p*<0.01). From 2008, at all sites, children older than five years were more frequently infected; the risk of being infected significantly increased with time, ranging from 0.37 to 1.50 in 2005 and from 2.03 to 5.10 in 2011 in this group (*p<*0.01). The risk of being *P. falciparum*-infected in children aged less than five years old significantly decreased from 2008 to 2011 (*p*<0.01).

**Conclusions:**

This study shows an increased risk of malaria infection in different areas of Gabon with over-five year-old children tending to become the most at-risk population, suggesting a changing epidemiology. Moreover, the heterogeneity of the malaria burden in the country highlights the importance of maintaining various malaria control strategies and redefining their implementation.

## Background

According to the level of malaria transmission and immunity acquisition, vulnerable populations differ in endemic areas. In highly endemic settings, children under five years and pregnant women are the most affected, constituting the main target population of new malaria control strategies as recommended by the World Health Organization (WHO) [1]. In countries that have adopted such strategies, a two- to five-fold decrease in malaria prevalence among febrile patients has been observed [2]- [5]. Nevertheless, 216 million malaria cases were still reported in 2011, mainly in sub-Saharan Africa, suggesting to maintain the targeted strategies [2]. An estimation of the real burden of the disease is needed to better identify populations at risk, and will contribute to adapt control strategies [6-8]. Mapping of malaria prevalence is one tool that provides unique opportunities to redefine regional *Plasmodium falciparum* risk distributions, and allows estimation of regional intensity of transmission, populations at risk, disease burden and level of prevention coverage [9-11]. According to the Malaria Atlas Project (MAP), baseline global evidence of malaria parasite prevalence, from reliable data, is essential to generate a current malaria world map; as such maps must be updated frequently [12]. However, clinical data from health care-seeking paediatric patients can be used by providing additional information to estimate malaria burden and to characterize the epidemiology of the disease [13].

In Libreville, the main urban city of Gabon, a reduction of malaria prevalence has been observed following implementation of new control strategies [14]. A trend towards a higher risk of *P. falciparum* infection in children aged more than five years was reported, indicating the need to implement appropriate malaria control strategies. Previous surveys performed several years ago across the country showed differences in prevalence of malaria-positive blood smears (MPBS) ranging from 24% to 59% (Kombila, unpublished data). With the support of the Global Fund, the Malaria National Control Programme (MNCP) organized the deployment of insecticide-treated bed nets (ITN) and artemisinin-based combination therapy (ACT) within the country. Indeed, in 2010, ITN coverage was 50% and ACT represented almost 99% of all antimalarial drugs prescribed in public health structures in Gabon [2]. The aim of the study was to estimate the clinical malaria burden among febrile children less than 11 years of age before and after six-year of deployment of malaria control strategies in different areas of Gabon.

## Methods

### Study site and population

Prospective cross-sectional surveys were conducted between 2005 and 2011 in Gabon, at Libreville the capital city and Melen one of its suburban area, at Port-Gentil the second largest town of the country and at Oyem. Recruitment was carried out at four health care centres: i) the Malaria Clinical and Operational Research Unit (MCORU) in Libreville, the capital city of Gabon; ii), the regional hospital of Melen (HREM), a semi-urban area located at 11 km north of Libreville; iii) the regional hospital of Oyem (CHRO), a rural northern area; and, iv) the regional hospital of Port-Gentil (CHRP), an urban area located on a peninsula on the west coast. The first three sites were selected as sentinel sites for malaria survey by the Malaria National Control Programme (MNCP). The CHRP was selected because previous data highlighted a low frequency of confirmed malaria cases in private health structures that were more frequented by patients than public health services in the city. The CHRP was therefore built by the Ministry of Health to facilitate access to health care for the low-income population. Oyem is a town surrounded by the forest with a level of urbanization being the lowest compared to Libreville, the capital city, Port-Gentil, the economic city, and Melen which benefits from its proximity to Libreville; people are living in villages located at less than one Km from the CHRO. Moreover, the main economic activities are related to agriculture and performed by the main population living at Oyem and in the surrounding areas. Indeed, workers in public administration are less represented.

As malaria transmission, predominantly caused by *P. falciparum* is perennial without significant fluctuation throughout the year in Gabon, the study team of MCORU collected data during at least one rainy and one dry season in each site. In 2011, during the study period, the recruitment at Oyem included out and inpatients, as well as febrile patients who were prescribed a biological analysis in private clinics since laboratory of the hospital was the only structure that could realize all the biological examinations at this time.

In sentinel sites, febrile patients are routinely screened for *P. falciparum* infection, therefore data collection was part of routine activities at the CHL, at Melen, and at Oyem where MCORU team was committed to set up the sentinel site activities according to WHO recommendation. At Port-Gentil, members of the study team took part in emergency and paediatric ward consultations and, during the study period, all the attending febrile children were screened for malaria.

Febrile paediatric outpatients and inpatients, aged less than 11 years old were included. Body temperature, history of fever, age, sex, bed net use, home treatment with anti malarial and location were collected. Data on self-medication have been collected through a detailed CRF. Patients were asked about previous history of fever and drug intake the month before the consultation, the type of molecule taken and the duration of the treatment during each survey throughout the study period.

### Malaria diagnosis

For each patient, a thick blood film was done according to the Lambaréné procedure and read as described previously [15]. Parasitaemia was expressed as a number of parasites per microliter of blood. Smears were considered negative after the examination of 100 oil immersion fields. Thin blood smears were used for species identification.

### Definition of febrile cases and *Plasmodium falciparum* infection episodes

A febrile case was defined for any child with axillary temperature ≥37.5°C or history of fever during the 48 hours prior to the day of consultation. Malaria case was defined by fever associated with a malaria positive blood smear (MPBS).

### Data analysis

All data were entered and cleaned using Epi-info version 3.3.2 (2005 CDC Atlanta). Analysis was performed using Statview 5.0 (SAS Institute, Cary, NC, USA). Proportion of malaria patients were compared using Pearson Chi-square and means or median by either Mann-Withney or Krushkal-Wallis non-parametric tests, according to site and time point as appropriate. Multiple comparisons of malaria frequencies according to site, age and year were performed by using a log rank test (Mantel Haenzel). A logistic regression analysis stratified by age groups (<5 years/>=5 years) was performed to assess the effect of site on malaria infection. The effect estimates are the odds ratio with a 95% confidence interval. Analyses resulting in values of *P*<0.05 were considered significant. All reported p-values are two-tailed. According to the sample size calculation, the number of patients recruited was always higher than the minimum required, although the total number of patients screened at Libreville and Port-Gentil in 2011 was lower compared to that of the other sites. Sample size was calculated taking into account previous prevalence of *P.falciparum* microscopic infection obtained in 2005 or 2008 according to the study areas. It was based on 5% precision with 95% level of confidence, using finite population correction according to NAIN (2006). Minimal sample size was 240 for Oyem, 199 for Libreville, 308 for Melen and 88 for Port-Gentil.

### Ethical approval

Malaria diagnosis, anaemia detection, drug resistance monitoring and interventions coverage in sentinel sites are the main strategies for malaria control of the Gabonese Ministry of Health (MH) represented by the Malaria National Control Program (MNCP). The Department of Parasitology-Mycology (DPM), is the reference laboratory for malaria diagnosis and anti-malarial drug resistance evaluation. The DPM is committed by the MH to carry out these evaluations throughout the country in order to provide reliable data for policy adjustment in collaboration with MNCP. All data obtained are part of routine activities in sentinel sites and at CHRP that are under the administrative supervision of the MH. Oral consent was obtained from parents or legal guardians for data publication. Each patient with a MPBS was treated according to national recommendations, using ACT, free of charge for children with uncomplicated malaria.

## Results

A total of 16,831 febrile children were enrolled: 78.5% (n=13,212) were less than five years old. Throughout the study period, the median age was the lowest in 2005, below 24.0 (12–48) months. In Libreville and Melen, the urban and semi-urban areas, the median age of the febrile patients did not vary between 2005 and 2011, although the IQR was larger in 2011 (Table 1). The sex ratio was of 1.2, comparable in all sites.

**Table 1:** Febrile patients, malaria infection and risk according to the site and the period of the survey

**Location**	**Year**	**N**	**Age, median (IQR**^**α**^**)**	**MPBS**^β^**n, (%)**	**Median age of infected patients, months (IQR**^**α**^**)**
Oyem	2005*	632	24[12–48]	302(47.8)	24[12–48]
Oyem	2008	689	24[12–48]	268(38.8)	36[17–48]
Oyem	2011	3,974	36[15–60]	1756(44.2)	36[19–72]
Libreville	2005	1,774	24[12–48]	495(27.9)	36[18–66]
Libreville	2008	4,684	24[12–48]	714(15.2)	40[18–72]
Libreville	2011	477	24[12–60]	115(24.1)	84[60–108]
Melen	2005	2,309	24[12–36]	833(36.1)	30[18–60]
Melen	2011	1,458	24[12–60]	456(31.3)	48[30–78]
Pog	2005**	559	15[9-26]	14(2.5)	26[17–45]
Pog	2011	275	24[13–48]	18(6.5)	60[32–96]
Total		16,831	24[12–48]		

^**α**^IQR: Interquartile ranges.

^β^MPBS: malaria positive blood smears.

*no patients above 5 years.

**only two patients had more than 5 years.

In 2011, bed net coverage ranged between 35% and 70% in all sites. Self-medication with anti-malarial drugs was of 22% in Libreville in 2008 and 18%, 9%, 20% and 10% in 2011 at Oyem, Libreville, Melen and Port-Gentil, respectively.

### *Plasmodium* infection prevalence

Based on parasitological diagnosis, frequencies of MPBS with *Plasmodium ovale* and *Plasmodium malariae* varied from 0% to 3% without variation during the study period. In contrast, throughout the study period, *P. falciparum* infection prevalence (29.5%; n=4,971/16,831) varied along the time. Between 2005 and 2008, a significant drop in malaria prevalence from 31.2% (n=1,644/5,274) to 18.3% (n=982/5,373), then an increase to 37.9% (n=2,345/6,184) in 2011 was observed (*p*<0.01). Statistical analysis showed a significant increase of malaria prevalence from 2008 to 2011 in Libreville, Port-Gentil and Oyem, whereas it slightly decreased from 2005 to 2011 at Melen (*p*<0.01) (Table 1).

### *Plasmodium falciparum* infections and age

The median age among the infected patients increased throughout the study period (*p*<0.01) (Table 1). From 2008, at all sites, children older than five years were more frequently infected compared to the youngest, with an odds ratio significantly higher for the older children (Table 2). The risk of being infected significantly increased throughout the years, ranging from 0.37 to 1.5 in 2005, and from 2.03 and 5.1 to 2011 (*p*<0.01). In 2011, it was the highest among children older than five years old at all sites compared to the previous years. Logistic regression showed a significant risk of being infected according to site and age (Table 3).

**Table 2:** febrile patients and malaria infection according to age and site

**Location**	**Year**	**< 5 years**		**> 5 years**		**OR for malaria in 5–10 years**	***p value***
		**n**	**IF**^**α**^**, %**	**n**	**IF**^**α**^**, %**		
Oyem	2005	632	47.8		ND*		
Oyem	2008	567	37.0	122	47.5	1.54 [1.04-2.28]	0.02
Oyem	2011	2,773	38.7	1201	56.3	2.03 [1.02-4.03]	0.05
Libreville	2005	1,347	32.1	427	14.7	0.37 [0.27-0.49]	<0.01
Libreville	2008	3,630	13.0	1054	23.0	2.00 [1.68-2.37]	<0.01
Libreville	2011	351	17.9	126	41.3	3.21 [2.06-5.02]	<0.01
Melen	2005	1,813	33.9	496	43.7	1.51 [1.22-1.83]	<0.01
Melen	2011	1,008	25.4	450	44.4	2.35 [1.86-2.97]	<0.01
Pog	2005	557	2.3	2	ND**		
Pog	2011	214	3.7	61	16.4	5.05 [1.9-13.44]	<0.01
Total		11,231	3062				

^α^IF: infected patients.

* no patients above 5 years.

**only two patients had more than 5 years.

**Table 3:** Logistic regression stratified by age groups (<5 years/>=5years)

	**< 5 years**				**>= 5 years**			
	**aOR**	**[95% Conf. Interval]**		**p**	**aOR**	**[95% Conf. Interval]**		**p**
Libreville	1.0	-	-	-	1.0	-	-	-
Melen	.8	.7	.9	0.01	1.6	1.4	2.0	<0.001
Oyem	1.4	1.2	1.7	<0.001	3.9	2.7	5.6	<0.001
Port-Gentil	10.8	9.6	12.3	<0.001	19.3	14.0	26.7	<0.001

Among children aged less than five years, a significant decrease of malaria cases was observed in most of the sites, except at Port-Gentil, during the study period (*p *<0.01) (Table 2). Indeed, the risk of being *P. falciparum*-infected in children aged below five years old significantly decreased from 3.16 (2.72-3.67) in 2008 to 1.45 (1.21-1.73) in 2011 (*p*<0.01).

### Parasite density distribution

Overall, the median parasite density (MPD) was 8,500 (1,400-43,555) P/μL. During the screening period, the MPD increased from 9,000 (1,433-41,548) P/μL to 14,700 (1,246-54,475) P/μL at Libreville and from 84 (70–168) P/μL to 5,600 (2,800-12,600) P/μL at Port-Gentil. Infected children from Oyem and Libreville carried the highest MPD: 15,085 (1,869-70,350) P/μL at Oyem and 6,300 (1,400-43,555) P/μL at Melen.

## Discussion

In areas with high level of malaria transmission, children under five years and pregnant women are the most vulnerable population and the main target of prevention strategies. The present study, carried out at four health care facilities in several areas of Gabon, estimated the evolution *P. falciparum* infection prevalence between 2005 and 2011. Large differences were observed between the sites, highlighting the heterogeneity of the epidemiological features of malaria in Gabon, even for sites that are geographically close together (e g, Libreville and Melen). Indeed, between, Melen and Libreville, the difference in the proportions of infected patients is lower probably due to the fact that health care facilities in both sites are accessible to all patients. Moreover, the difference between the patients consulting at both structures when considering the socioeconomic level is not so obvious, although, Melen is a sub-urban area of Libreville characterized by a majority of slums with a low income population, compared to the population of Libreville that is more complex in regards to the different living conditions.

As already described in urban cities, the prevalence was lower in coastal urban areas of Libreville and Port-Gentil. It was much higher at Libreville, probably due to a difference in urbanization level, equipment, housing and access to treatment between both cities. These differences do not seem to be related to ACT self medication and bed net coverage as reported elsewhere [16]. In all health care structures, ACT is the main treatment prescribed and frequency of self medication varied among sites, it was not associated with the proportion of MPBS. Moreover, despite bed net coverage variation in 2011, children less than five years are the main bed net users [2]. The confirmed low frequency of malaria infection in Port-Gentil compared to Libreville, although a high EIR and risk of transmission as reported by Mourou *et al.*[17] could be related to the health care management of the majority of the population in Port-Gentil. Indeed, a great proportion of people benefit for free medical care provided at private health structure of oil companies where more than 70% of the active population work, each family having care free of charge; furthermore, presumptive treatment of fever with anti-malarial drugs is the rule in these centers.

Previous studies reported a decline in malaria prevalence in febrile children over time [4,14,18,19]. In Mlomp, Senegal, an area of moderate malaria transmission, the risk of malaria decreased by about 32 times between 1996 and 2010, including the control strategy implementation period [18]. A reduction of malaria case frequency was observed among inpatients hospitalized in Libreville between 2002 and 2008, accompanied by a high frequency of viral and bacterial infections [20]. The decline of *P. falciparum* infection rates already observed in Libreville, is also confirmed in other areas of the country [14].

After the decline in the proportion of malaria cases at all sites between 2005 and 2008, a rebound in infection rates in rural and urban areas appears. In the same time, a slowdown in the prevention activities led by the MNCP and a low ITN coverage may partly explain this phenomenon. As an example, in Malawi, from 2001 to 2005, the proportion of malaria cases decreases of 50% (34.5% to 17.1%), followed by a rebound to 25%. An association with the slowing of interventions from 2001 to 2010 is characterized by a reduction of free ITN distribution, a limited free access to ACT in health care facilities for vulnerable populations, the lack of re-impregnation of used bed nets and indoor residual spraying (IRS) [16]. In Kenya, a higher child mortality attributable to malaria was also associated to a drop in the stock of essential anti-malarial drugs and a disruption of services during civil unrest [21]. In Zanzibar and Zambia where interventions are maintained, there is no rebound in malaria morbidity and mortality [22,23]. Others factors such as vector resistance to insecticide could also contribute to increase malaria prevalence. In Port-Gentil and Libreville, the main vectors of *Plasmodium* carry a high proportion of molecular markers of resistance to the commonly used insecticides, with an impact on the effectiveness of the current vector control programs [17]. In Zambia, both *Anopheles gambiae s.s*. and *Anopheles funestus s.s*. were controlled effectively with the ITN and IRS programme, maintaining a reduced disease transmission and burden [24]. Environmental factors such as a slower urbanization of Melen and Oyem, as well as a probable high entomological inoculation rate (EIR) could all explain the slow reduction of malaria cases in these sites.

Consistent with other tropical areas, children under five years constitute the majority of patients consulting for fever [25]. The proportion of infected children under five years globally decreased throughout the survey in each site, except at Port-Gentil where it increased probably due to the EIR, and underlying a difference of exposure of patients in both age groups. Between 2005 and 2011, the reduction of malaria cases among children under five years old was of 30% at Melen and almost 50% at Libreville. An obvious shift in the age of infected children towards those aged over five years and who are at higher risk of being infected, confirms previous data obtained from 28,000 children [14]. Malaria risk increased from 0.37 to 5.05 over time among older children during the study period, highest in urban areas, Port-Gentil and Libreville, suggesting a delay in the immunity acquisition as observed in areas with lower malaria transmission [26,27]. Another explanation could be the density of *Anopheles* population in both cities [17,28]. In Thies, an urban area of Senegal, the most affected population was aged from four to 20 years old [29]. In Tanzania, where malaria prevalence usually peaks in younger children (23–25 months), an increased risk of 1.7 of infection was found for children five to 13 years old compared to those of six months to four years [30]. The younger children were also significantly more likely to sleep under ITNs compared to those aged from five to 13 years [31]. Similar trends were found in both rural and urban areas in Uganda [19].

Together, the rates of malaria prevalence and the shift in the age of at risk children evoke a changing epidemiology of malaria in Gabonese cities, which cannot be all considered as malaria hyperendemic areas. Although the transmission is perennial, it rather becomes meso-endemic in some towns such as Libreville and it is much lower at Port-Gentil. Cohort studies and new entomological surveys from other areas are urgently needed to further characterize the malaria transmission in the country.

This study has some limitations. Patients could not be screened at all the sentinel sites. Otherwise, this study is a health centre survey. Therefore, it does not represent the situation in the whole population but it already provides reliable important data. Furthermore, assessment of bed net effective use and type (i e, insecticide treatment or re-impregnation), and their direct impact on malaria prevalence cannot be established.

## Conclusions

Baseline epidemiological data are necessary to develop appropriate malaria control strategies and public health policy. This study shows that despite a decline of malaria cases observed in several endemic areas of Gabon, there is an increased risk of malaria infection among children older than five years who are becoming the most at-risk population. The heterogeneity of malaria epidemiology in Gabon underlines the need to increase, to maintain the various malaria control strategies and to redefine their implementation on a continual basis, particularly in urban areas where a rebound in malaria is being observed. Further entomological surveys in rural and semi-urban areas are needed in order to better support malaria prevention and control. This work has important implications for the planning of future campaigns based on the distribution of long-lasting, insecticide-treated nets and the introduction of intermittent preventive treatment for children in Gabon

## Competing interests

The authors declare that they have no competing interests.

## Authors’ contributions

The study was initiated by MK. DPMM and MKBA conducted the study with the contribution of all authors. DPMM and MKBA interpreted the data and drafted the manuscript. Data were analyzed by MKBA and EK. NJ, MOM, MJR and MCRU team participated to the field study. All authors revised and approved the final manuscript.
